# C1 Metabolism Inhibition and Nitrogen Deprivation Trigger Triacylglycerol Accumulation in *Arabidopsis thaliana* Cell Cultures and Highlight a Role of NPC in Phosphatidylcholine-to-Triacylglycerol Pathway

**DOI:** 10.3389/fpls.2016.02014

**Published:** 2017-01-04

**Authors:** Coline E. Meï, Mathilde Cussac, Richard P. Haslam, Frédéric Beaudoin, Yung-Sing Wong, Eric Maréchal, Fabrice Rébeillé

**Affiliations:** ^1^Laboratoire de Physiologie Cellulaire Végétale, UMR 5168 CNRS – CEA – INRA – Université Grenoble Alpes, Bioscience and Biotechnologies Institute of GrenobleCEA-Grenoble, Grenoble, France; ^2^Department of Biological Chemistry and Crop Protection, Rothamsted ResearchHarpenden, UK; ^3^Département de Pharmacochimie Moléculaire, UMR 5063 CNRS – Université Grenoble AlpesGrenoble, France

**Keywords:** triacylglycerol synthesis, phosphatidylcholine synthesis, non-specific phospholipase C, C1 metabolism, methylation index, nitrogen deficiency, *Arabidopsis*

## Abstract

Triacylglycerol (TAG) accumulation often occurs in growth limiting conditions such as nutrient deprivations. We analyzed and compared the lipid contents of *Arabidopsis* cells grown under two conditions that inhibited growth as a way to study interactions between membrane and storage lipids. In order to inhibit C1 metabolism, the first condition utilized methotrexate (MTX), a drug that inhibits methyl transfer reactions and potentially reduces Pi-choline synthesis, the polar head of phosphatidylcholine (PC). MTX-treated cells displayed a 10- to 15-fold increase in TAG compared to that found in control cells. This corresponded to a net increase of lipids as the total amount of membrane glycerolipids was minimally affected. Under this condition, PC homeostasis appeared tightly regulated and not strictly dependent on the rate of Pi-choline synthesis. The second condition we investigated involved nitrogen deprivation. Here, we observed a 40-fold increase of TAG. In these cells, the overall lipid content remained unchanged, but membrane lipids decreased by a factor of two suggesting a reduction of the membrane network and a rerouting of membrane lipids to storage lipids. Under all conditions, fatty acid (FA) analyses showed that the FA composition of TAG was comparable to that in PC, but different from that in acyl-CoA, suggesting that TAG accumulation involved PC-derived DAG moieties. In agreement, analyses by qPCR of genes coding for TAG synthesis showed a strong increase of non-specific phospholipase C (*NPC*) expressions, and experiments using labeled (fluorescent) PC indicated higher rates of PC-to-TAG conversion under both situations. These results highlight a role for NPC in plant cell oil production.

## Introduction

In all organisms glycerolipids are essential components of the membrane architecture and the main form of storage lipids. Membrane glycerolipids are diesters of fatty acids (FA) and glycerol, the third hydroxyl function of the glycerol backbone being linked with a polar group, whereas storage glycerolipids are triesters of FA, i.e., triacylglycerol (TAG). These storage lipids represent a highly reduced form of carbon that serve as energy reserves, constituting an important resource to meet the demands for food, biofuel, green chemistry, or many other industrial applications (Lu et al., 2011).

**FIGURE 1:**
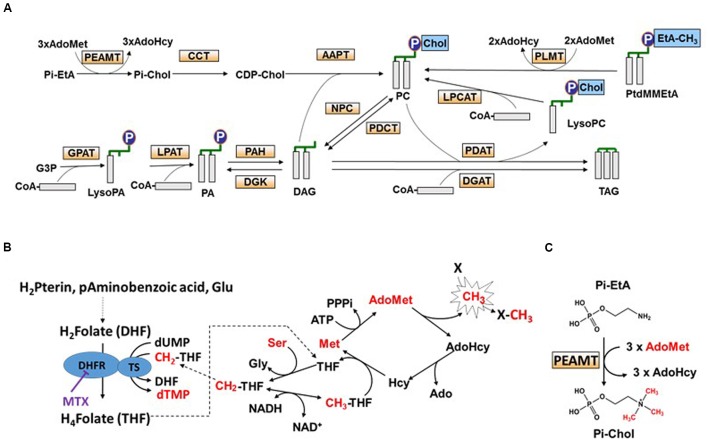
**Tetrahydrofolate (THF) synthesis, C1 metabolism, and triacylglycerol (TAG) biosynthesis in plant cells. (A)** Scheme representing phosphatidylcholine (PC) and TAG biosynthesis pathways, and the interconversion between PC and TAG. CDP-Chol, CDP-choline; LysoPC, lyso-phosphatidylcholine; PtdMMEtA, phosphatidylmonomethylethanolamine; G3P, glycerol-3-Pi. PEAMT, phosphoethanolamine *N*-methyltransferase; CCT, CTP:Pi-choline cytidylyltransferase; AAPT, DAG:CDP-choline phosphotransferase; GPAT, glycerol-3-Pi acyltransferase; LPAT, lysophosphatidic acid acyltransferase; PAH, phosphatidic acid hydrolase; DGK, diacylglycerol kinase; NPC, non-specific phospholipase C; PDCT, phosphatidylcholine:diacylglycerol cholinephosphotransferase; DGAT, diacylglycerol acyltransferase; PDAT, phospholipid diacylglycerol acyltransferase; LPCAT, lyso-phosphatidylcholine acyltransferase; PLMT, phospholipid *N*-methyltransferase. **(B)** The pathway of THF synthesis and the methyl cycle. In bikont organisms (such as plants) dihydrofolate reductase (DHFR) is a bifunctional enzyme, supporting both DHFR and thymidylate synthase (TS) activities, whereas in unikont organisms (such as animals) DHFR is mono-functional. Methotrexate (MTX) is a structural analog of DHF that specifically inhibits the DHFR domain. Inhibition of the DHR domain impairs cell division and the methyl cycle activity (methylation reactions). Methyl groups are transferred from methionine (Met) to an acceptor X *via* AdoMet, the universal methyl donor, and then Met is regenerated from serine and THF. H2Pterin, dihydropterin; Glu, glutamate; H_2_Folate or DHF, dihydrofolate; H_4_Folate or THF, tetrahydrofolate; CH_2_-THF, methylenetetrahydrofolate. CH3-THF, methyltetrahydrofolate; AdoMet, *S*-adenosylmethionine; AdoHcy, *S*-adenosylhomocysteine; Ado, adenosine; Ser, serine; Gly, glycine. **(C)** The synthesis of Pi-choline from Pi-ethanolamine. Pi-choline is the precursor of PC. Methyltransferases such as PEAMT are controlled by the AdoMet/AdoHcy ratio. Pi-Chol, Pi-choline; Pi-EtA, Pi-ethanolamine.

The balance between the various species of membrane glycerolipids and their relationships with storage glycerolipids are complex and not fully understood. Diacylglycerol (DAG) and phosphatidic acid (PA) are the precursors of all glycerolipids (Li-Beisson et al., 2013), i.e., galactolipids in plastids and phospholipids in other membranes. Neosynthesis of DAG occurs either in the endoplasmic reticulum (ER) or in plastids. This involves the sequential addition of two acyl chains from the acyl-ACP in plastids or the acyl-CoA pool in the ER to the *sn*-1 and *sn*-2 positions of glycerol-3-Pi (*sn* is for stereospecific number) to form PA (Kim et al., 2005; Joyard et al., 2010; Shockey et al., 2016). PA is then dephosphorylated generating DAG (Nakamura et al., 2007, 2009). Alternatively, DAG can also be produced from a phospholipid [e.g., phosphatidylcholine (PC)] by the removal of its polar head Pi-choline (Bates, 2016). Polar head removal can occur via multiple reactions (**Figure 1A**): (i) the reverse action of a DAG:CDP-choline phosphotransferase (AAPT), the enzyme involved in PC synthesis (Slack et al., 1983); (ii) a phosphatidylcholine:diacylglycerol cholinephosphotransferase (PDCT), an enzyme that can reversibly transfer a Pi-choline from PC to DAG (Lu et al., 2009); or, (iii) a non-specific phospholipase C (NPC) that can remove the Pi-choline head from PC (Nakamura et al., 2005).

Several pathways can be involved for the synthesis of storage lipids, i.e., TAG, which are assembled/produced in the ER by acylation of DAG at the *sn*-3 position (see **Figure 1A**). The first route is referred to as the Kennedy pathway and requires three sequential acylations of the glycerol backbone from the acyl-CoA pool. The last acylation is catalyzed by an acyl-CoA dependent diacylglycerol acyltransferase (DGAT), which adds a third FA from the acyl-CoA pool to DAG.

Although there are at least four different genes related to DGAT in higher plants (Lu et al., 2011), only type 1 and type 2 functions have been studied. Whereas DGAT1 plays a major role controlling TAG accumulation in seeds, DGAT2 appears to prefer substrates with unusual FA (hydroxy-, epoxy- or conjugated FAs; Burgal et al., 2008; Chen and Smith, 2012; Li et al., 2012). Another route to produce TAG involves a phospholipid:diacylglycerol acyltransferase (PDAT) activity (Bates and Browse, 2011), where the FA moiety added at the *sn*-3 position of the glycerol backbone originates from a phospholipid (mostly PC), and not from the acyl-CoA pool (Dahlqvist et al., 2000) (**Figure 1A**). The resulting lyso-PC is then recycled back into PC by a lyso-phosphatidylcholine:acyltransferase (LPCAT1, LPCAT2; Xu et al., 2012; Lager et al., 2013).

Phosphatidylcholine is known to be the major site of ‘acyl editing.’ In this process, FA from PC are exchanged with FA from the acyl-CoA pool through a rapid deacylation-reacylation cycle (Bates et al., 2012; Bates, 2016). Through this cycle, oleic acid (18:1) synthesized in plastids and transferred in the cytosol as oleyl-CoA is attached to lyso-PC, and then converted to polyunsaturated FA (PUFA) by FA desaturases. PUFA are thereafter released from PC in the acyl-CoA pool by a phospholipase A cleavage or the reverse action of LPCAT, to be eventually available for other glycerolipid syntheses, including TAG (Li-Beisson et al., 2013; Bates, 2016). Kinetic labeling experiments suggest a channeling of newly synthesized acyl chains toward PC, and indicate that PC acyl editing is the main pathway for the initial incorporation of FA into glycerolipids (Bates and Browse, 2012; Tjellstroem et al., 2012). In other words, the newly synthesized acyl chains are first addressed to PC, desaturated and then released back from PC into the acyl-CoA pool. Thus, the FA composition of DAG and its downstream product TAG, may differ depending on the metabolic route followed to produce DAG: (i) *de novo* synthesis using newly synthesized 18:1-CoA; (ii) *de novo* synthesis using PC-edited acyl-CoA; or (iii) PC-derived DAG (Bates and Browse, 2012). In addition, as described above, the third FA present at the *sn*-3 position of the glycerol backbone may also have different origins, i.e., the acyl-CoA pool or PC.

Thus, PC appears as a metabolic platform in the general scheme of glycerolipid syntheses, draining most of the acyl fluxes for their maturation by addition of double bonds and their redistribution toward the various glycerolipids. The main route for the biosynthesis of PC occurs in the ER and involves the condensation of DAG and CDP-choline, a reaction catalyzed by AAPT (also called DAG-CPT). CDP-choline is synthesized from Pi-choline by a CTP:Pi-choline cytidylyltransferase (CCT; Inatsugi et al., 2009; Li-Beisson et al., 2013), and Pi-choline, in turn, is formed by three sequential methylations of phosphoethanolamine (**Figures 1A,C**). These methylations are catalyzed by a phosphoethanolamine *N*-methyltransferase (PEAMT) and require *S*-adenosylmethionine (AdoMet) as methyl donor. AdoMet, in turn, relies on methionine (Met) and on the tetrahydrofolate (THF) cofactor for its synthesis (**Figure 1B**). In *Arabidopsis*, three separate genes for PEAMT can be found. PEAMT1 and three catalyze the three methylation steps, whereas PEAMT2 catalyzes only the last two (BeGora et al., 2010). Several studies indicate that PC synthesis is tightly controlled at the level of PEAMT, either through feed-back inhibition by Pi-choline (Witola and Ben Mamoun, 2007) or by PA (Carman and Henry, 2007; Jost et al., 2009; Eastmond et al., 2010). Another metabolic route which potentially involves PC synthesis is the AdoMet-dependent methylation of phosphatidylmonomethylethanolamine, a reaction catalyzed by a phospholipid *N*-methyltransferase (PLMT). This second route, however, appears to have only minor contribution to PC synthesis in plants (McNeil et al., 2000; Keogh et al., 2009).

Triacylglycerol content may fluctuate widely depending on the growth conditions or the specificity of the tissue. Oilseeds generally contain large amount of storage lipids, and the reactions involved in TAG synthesis have been mostly studied in these tissues [for a recent review, see (Bates, 2016)]. In non-seed tissues, such as mesophyll cells or microalgae, the amount of TAG is generally low but can increase as part of a response to particular situations. The best documented situations concern nutrient deficiencies in microalgae. Indeed, it was shown that nitrogen and phosphate deficiencies, the most often limiting or co-limiting nutrients in oceans, trigger important lipid remodeling associated with strong accumulations of TAG (Abida et al., 2015). These observations are at the basis of numerous strategies to produce third generation biofuels. These effects have been less studied in higher plants, but it was observed in *Arabidopsis* seedlings that the amount of TAG increased during nitrogen deprivation (Gaude et al., 2007; Shimojima et al., 2015), and that such increase was also significantly enhanced in the presence of sugar (Yang et al., 2011). The reasons why nutrient deficiencies induce TAG accumulation are not clear. It is possible that in the absence of cell division the carbon precursors normally used for protein synthesis or energy metabolism are partly redirected toward TAG production (Singh et al., 2011; Simionato et al., 2013; Yang et al., 2013). From this point of view, it could be possible that any situation that affects growth rate and cell division triggers TAG accumulation. In support to this hypothesis, it was shown in yeast that TAG synthesis and degradation fluctuate according to the cell division cycle (Kurat et al., 2006; Zanghellini et al., 2008; Kohlwein, 2010). However, the impact of growth limiting conditions upon the expression of genes involved in lipid synthesis remains to be investigated, together with the pathway involved in the production of storage lipids.

In the present article, we evaluated the glycerolipid composition and the expression of genes related to TAG synthesis in suspension cultures of *Arabidopsis* mesophyll cells in different situations that affect growth and cell division. We compared the effects of different chemical inhibitors, 5-fluorouracile (5-FU), an inhibitor of thymidylate synthase, taxol, an inhibitor of microtubule disassembly, and methotrexate (MTX), an antifolate drug that blocks nucleotide synthesis and also inhibits the methyl transfer reactions such as those involved in Pi-choline synthesis. Such a ‘chemogenomic’ approach has proven successful in several studies aimed to better understand lipid metabolism in plants and microalgae (Botte et al., 2011; Boudiere et al., 2012; Franz et al., 2013). Then we focused on MTX and compared its effects with a nitrogen-deficient situation, a condition often encountered by plants and algae and known to induce large amounts of TAG in microalgae.

## Materials and Methods

### Culture Conditions

*Arabidopsis thaliana* cells (ecotype Columbia) were grown as suspension cultures in 200 mL Murashige and Skoog medium (MSP09-50LT, Caisson Laboratories, Inc, USA) or in Murashige and Skoog medium devoid of nitrogen (MSP07-10LT, Caisson Laboratories, Inc, USA) and supplemented with 1.5% (w/v) sucrose, 1.2 mg l^-1^ of 2,4-dichlorophenoxyacetic acid, 50 mg.l^-1^ of monobasic potassium phosphate, and 39 mg.l^-1^ of dibasic sodium phosphate. Cultures were kept under continuous light (100 μE m^-2^ s^-1^) at 22°C and agitated with rotary shaking at 125 rpm. Cells were maintained by a 1:9 (culture:fresh media) split every 7 days. Cell growth was monitored by measuring the fresh weight: 5 mL of the culture were removed, filtered on glass microfiber filters (Whatman) and weighted.

### Incubation Experiments

*Arabidopsis thaliana* cells were subcultured as described above 48 h prior starting the incubation experiment. After 48 h, the cells were subcultured again at a concentration of 3 g of cells per 100 mL in fresh MS medium containing or not nitrogen, and let to recover for 5 h. This procedure allows the culture to be in exponential growth phase at the beginning of the experiment. Then, for the nitrogen deficiency experiment, the cells were washed three times in a MS medium deprived of nitrogen (MSP09-50LT, Caisson Laboratories, Inc, USA) and let to grow in the same nitrogen-deprived medium for 72 h. For the experiments using inhibitors, chemicals (all from Sigma–Aldrich) were then added to the cell culture: MTX 3 μM, in 100% Tris 50 mM (final Tris concentration in cell culture: 0.6%), 5-formyltetrahydrofolate 50 μM, Methionine 2 mM, Pi-Choline 500 μM, 5-fluorouracil 200 μM in ultra-pure water and taxol (Paclitaxel) 50 μM in DMSO (final DMSO concentration in cell culture: less than 0.1%). Control cells received the equivalent volume of Tris 50 mM or DMSO. Cell growth was monitored every day. After 72 h of incubation, cells were filtered, weighed and frozen in liquid nitrogen for lipid and gene expression analyses. Experiments were performed 3–5 times for each condition.

### Lipid Analysis

Lipid extraction and analysis were performed as previously described (Simionato et al., 2013; Abida et al., 2015). Briefly, 0.5 g of freeze-dried cells were suspended in 4 mL of boiling ethanol for 5 min to prevent lipid degradation, and lipids were extracted by addition of 2 mL methanol and 8 mL chloroform at room temperature. After filtration through glass wool, cell debris were rinsed with 3 mL chloroform/methanol 2:1 (v/v) and 5 mL of 1% NaCl were then added to the filtrate to initiate biphase formation. The chloroform phase was collected and dried under argon before solubilizing the lipid extract in pure chloroform. Total glycerolipids were quantified from their FAs after transformation as methyl esters (FAME), and analyzed by a gas chromatography-flame ionization detector (GC-FID; PerkinElmer) on a BPX70 (SGE) column, as thoroughly described elsewhere (Jouhet et al., 2003). FAME were identified by comparison of their retention times with those of standards (Sigma) and quantified by the surface peak method using 15:0 or 21:0 for calibration. To quantify the various classes of glycerolipids, lipids were separated by thin layer chromatography (TLC) onto glass-backed silica gel plates (Merck) using two distinct resolving systems for polar and neutral lipids, as previously described (Jouhet et al., 2003). Then, they were visualized under UV light, after spraying with 2% 8-anilino-1-naphthalenesulfonic acid in methanol, and scraped off the plate. Lipids were recovered from the silica powder after addition of 4 mL chloroform:methanol 1:2 v/v, thorough mixing and addition of 8 mL chloroform and 3.2 mL H2O and collection of the chloroform phase (Bligh and Dyer, 1959). Lipids were then dried under argon and quantified after methanolysis by GC-FID as described above.

Extraction and quantification were performed from 4 to 5 independent biological repeats for the MTX condition, three to five independent biological repeats for MTX + Met and three independent biological repeats for MTX + Pi-choline conditions. To correct inherent variations from one culture to another (Meï et al., 2015), a control was systematically run for each experiment and data were analyzed as fold change versus the corresponding controls. Quantifications were normalized by multiplying the experiment/control ratio by the average value of the control. Statistical analyses were performed with GraphPad Prism software using unpaired *t*-test with α = 5%.

### Acyl-CoA Analysis

Acyl-CoAs were extracted as previously described (Larson and Graham, 2001) from frozen freeze-dried cell culture samples (10 mg d.wt.) and analyzed using LC-MS/MS + MRM in positive ion mode. The LC-MS/MS + MRM analysis (using an ABSciex 4000 QTRAP Framingham, MA, USA) was performed as described [Haynes et al., 2008; Agilent 1200 LC system; Gemini C18 column (Phenomenex, Torrance, CA, USA), 2 mm inner diameter, 150 mm length, particle size 5 μm]. For the identification and calibration, standard acyl-CoA esters with acyl chain lengths from C14 to C20 were purchased from Sigma as free acids or lithium salts.

### AdoMet, AdoHcy, and Pi-Choline Determinations

Cells, about 0.2–0.5 g were frozen and ground in liquid nitrogen using a mortar and a pestle. Grinded cells were allowed to thaw in the presence of 300 μl of acetonitrile/acetic acid 0.5% (50:50, v/v). The cell suspension was centrifuged for 10 min at 14000 rpm (Eppendorf) to remove cell debris, and the pellet was extracted a second time with the acetonitrile/acetic acid mixture. The metabolites were quantified using a LC (Agilent 1100 series)/MS (LTQ, Thermofisher) device. The reverse phase chromatographic conditions were: column (Kinetex C18, 2.6 μm, 100 mm × 3 mm, Phenomenex); isocratic elution with methanol 20% in acetic acid 0.5%, flow rate 0.2 ml/min. Pi-choline was identified from its retention time (2.2 min) and by the MS2 signal corresponding to the loss of the Pi group (parent ion m/z 184.1, product ion m/z 86.1). AdoMet was identified from its retention time (3 min) and by the MS2 signal corresponding to the loss of the Met group (parent ion m/z 399.1, product ion m/z 250.1). AdoHcy was identified from its retention time (2.7 min) and by the MS2 signal corresponding to the loss of the Hcy group (parent ion m/z 385.4, product ion m/z 250.1). Quantification were performed from at least three independent biological repeats for each condition. Statistical analyses were performed with GraphPad Prism software using unpaired *t*-test with α = 5%.

### Quantitative RT-PCR

Real-time quantitative RT-PCR experiments were performed using cDNA synthesized from total RNA isolated from control and treated *Arabidopsis* cells, according to the manufacturer instructions (RNeasy Plant Mini Kit, Qiagen). Amplification of contaminating DNA was prevented by DNAse treatment of RNA samples. Specific primer sequences were designed for each tested gene (Supplementary Table 2). The real-time PCR reactions were carried out on a Rotor-Gene 3000 instrument (Corbett Research) using SYBR Green JumpStart Taq ReadyMix (Sigma–Aldrich). Quantification of gene expression was performed using the comparative Ct method within the Rotor-Gene 3000 Software. Each data were normalized with three reference genes chosen for their absence of variation in the MTX and nitrogen-deprived conditions (*ACT7*–At5g09810; *UBQ5*–At3g62250; *ARP1*–At1g43170). Each value represents the average of three independent biological repeats, each repeat being analyzed in triplicates. Statistical analyses were done with GraphPad Prism software using unpaired *t*-test and the Holm–Sidak method with α = 5%.

### Laser Scanning Confocal Microscope

Cells, 120 μL, were diluted two times in MS media. Image acquisitions were performed on a LSCM (Leica, TCS SP2) and an Apo 45x∖1.25–0.75 oil immersion objective lens. For Nile red staining, cells were incubated for 20 min with 80 μL of Nile Red staining solution (2.5 μg ml^-1^ in DMSO, Sigma–Aldrich). Light emission corresponding to the Nile red signal was collected from 559 to 603 nm after excitation at 543 nm. Light emission corresponding to the Bodipy^®^ signal was collected from 500 to 535 nm after excitation at 488 nm.

### Fluorescent Dye-Labeled PC Experiments

Fluorescent labeled PC [TopFluor^®^ PC, 1-palmitoyl-2-(dipyrrometheneboron difluoride)undecanoyl-*sn*-glycero-3-phosphocholine] was purchased from Avanti Polar Lipids, Inc. Fluorescent FA (Bodipy^®^FL C16, 4,4-Difluoro-5,7-Dimethyl-4-Bora-3a,4a-Diaza-s-Indacene-3-Hexadecanoic Acid) was purchased from Thermo Fisher Scientific. Labeled standard DAG was obtained from digestion of TopFluor PC by phospholipase C (Sigma). Five hundred microliters buffer (50 mM Tris pH 8, 10 mM CaCl_2_, 0.4 mg ml^-1^ BSA) containing 10 μM TopFluor PC were incubated 2 h at 37°C in the presence of 10 units of phospholipase C. Then, DAG was recovered by the successive addition of 875 μl of chloroform:methanol 1:2 v/v, 625 μl H_2_O, 625 μl CHCl_3_. After centrifugation, the lower organic phase is collected and dried. Labeled standard TAG were obtained following a reaction of esterification between the fluorescent FA and (*S*)-1,2-dioleoyl-*sn*-glycerol (Sigma). (*S*)-1,2-Dioleoyl-*sn*-glycerol and fluorescent FA were dissolved in dichloromethane and 1-ethyl-3-(3-dimethylaminopropyl)carbodiimide hydrochloride (EDCI) was added with a catalytic amount of 4-dimethylaminopyridine (DMAP). The mixture was stirred overnight at room temperature. Fluorescent TAG was isolated by flash chromatography (silica gel, 20% cyclohexane/dichloromethane). For cell labeling experiments, cells were grown in the presence of 500 nM TopFluor^®^ PC dissolved in pure ethanol (final concentration of ethanol 0.05%) for 3 days with or without MTX or nitrogen as described above. Then, cells were rinsed three times with fresh media to remove the extra TopFluor^®^ PC before collection for confocal microscopy and lipid extraction. Neutral lipids were separated by TLC, as described above, and compared for their migration with labeled standards. Fluorescence of labeled lipids was measured at 503 nm following excitation at 488 nm (ChemiDoc Imaging System, Biorad). Pixels were quantified using the ImageLab software (BioRad).

## Results

### Growth Inhibition and TAG Accumulation

**FIGURE 2:**
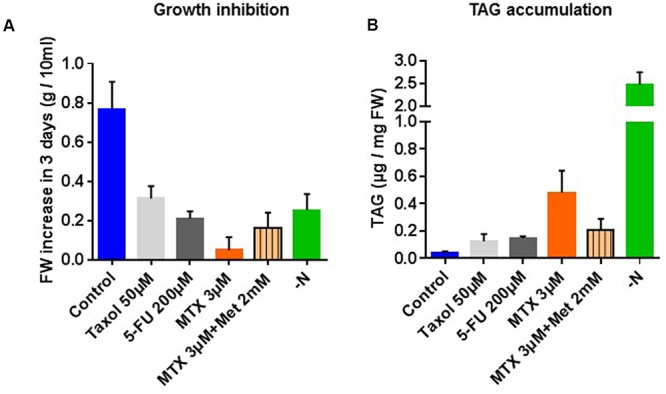
**Growth and TAG content of *Arabidopsis thaliana* cell cultures incubated 72 h in the presence of taxol, 5-fluorouracile (5-FU), MTX, MTX+Met or grown in a nitrogen-deficient medium. (A)** Fresh weight after 72 h of treatments. The initial cell concentrations at *t* = 0 h were 0.3 g per 10 mL of culture in each conditions. **(B)** TAG content expressed as μg of fatty acid (FA) per mg of fresh weight. All the measurements were made after 72 h of treatment. The data are means ± SD of five (MTX and MTX+Met) or three (Taxol, 5-FU, and –N) independent biological repeats.

Cell suspension cultures of *Arabidopsis* are useful tools to study the effects of nutrients or drugs on cell physiology because their concentrations in the external medium can be easily controlled. It must be noted that, even if the glycerolipid composition (distribution) is relatively robust in these cells, variations in the total amount of lipids can be observed from one culture to another (Meï et al., 2015). Thus, data from each assay were always compared to a control originating from the same culture batch as the treated cells (see Materials and Methods section). The idea that TAG accumulation is connected with the growth rate is often encountered in the literature, but there is no detailed analyses about how this affects the overall lipid composition in higher plants. We tested the effects of 5-FU an inhibitor of thymidylate synthesis (Neuburger et al., 1996), MTX an inhibitor of THF synthesis (Ravanel et al., 2011), taxol an inhibitor of microtubule disassembly (Kudo et al., 2014), and nitrogen deficiency. As shown in **Figure 2A**, all these treatments resulted in a significant inhibition of growth after 3 days, MTX being the strongest inhibitor (see also the Supplementary Figure 1 for the corresponding growth curves). In all these experiments TAG accumulated (**Figure 2B**), suggesting some pleiotropic effect associated with these stress conditions. However, TAG did not accumulate to the same extent in all situations, also suggesting some specific effects on lipid metabolism. Indeed, whereas TAG contents increased only three to four times in the presence of 5-FU or taxol, MTX-treated cells and cells grown in the absence of nitrogen displayed, respectively, 13 and 40 times more TAG than in control cells.

Methotrexate is a specific inhibitor of dihydrofolate reductase (DHFR), a key enzyme in THF synthesis (Ravanel et al., 2011). THF is the central cofactor of C1 metabolism, which is involved in nucleotide and methionine syntheses (**Figure 1B**). Met, in turn, is the precursor of *S*-adenosylmethionine (AdoMet), the universal methyl donor. Consequently, the *S*-adenosylmethionine/S-adenosylhomocysteine ratio (AdoMet/AdoHcy; also called methyl index) decreases (Loizeau et al., 2007; Van Wilder et al., 2009), and most of the methyl transfer reactions such as those involved in Pi-choline and PC syntheses are strongly lowered (**Figures 1B,C**). When Met was provided together with MTX in order to reactivate the methyl cycle activity, some growth was restored (**Figure 2A**) and the observed TAG accumulation reached approximately the level recorded with 5-FU or taxol (**Figure 2B**), thus suggesting that the accumulation recorded with MTX alone was at least partly due to the inactivation of methyl transfer reactions.

The relationship between nitrogen and lipid metabolisms is not fully understood. In contrast with the MTX condition, nitrogen deficiency is a physiological situation often encountered and to which plants have adapted. One of the adaptive answers is to accumulate TAG, possibly to dispose of a source of energy readily available when the growth conditions subsequently gets better.

To better understand the mechanisms underlying TAG accumulation we analyzed in details the lipid composition and the expression of related genes in these two case studies.

### Lipid Composition and PC Homeostasis in MTX Treated Cells, Blocking C1 Metabolism

**FIGURE 3:**
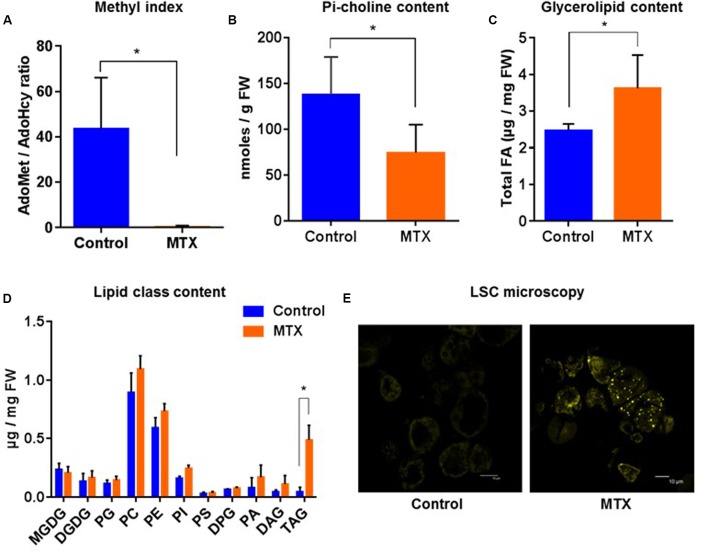
**Pi-choline content, methyl index, and lipid analysis of *A. thaliana* cells incubated 72 h in the presence of MTX. (A)** AdoMet/AdoHcy ratio. **(B)** Pi-choline concentration. **(C)** Total glycerolipid content expressed as μg of FA per mg of fresh weight. **(D)** Quantitative analysis of the various glycerolipids (expressed as μg of FA per mg of fresh weight). **(E)** Nile-Red staining and LSC microscopy imaging showing an accumulation of lipid droplets in MTX treated cells. All the measurements were made after 72 h of treatment. The data are means ± SD of five independent biological repeats for each condition. Significant differences (*P* < 0.05) are shown by an asterisk and were calculated by an unpaired multiple *t*-test using GraphPad Prism software.

As a first case study, MTX-treated cells displayed an AdoMet/AdoHcy ratio that was strongly reduced compared to control cells (from 40 to 0.4; **Figure 3A**), illustrating the blocking of C1 metabolism. As a result, the Pi-choline content was lowered by about a factor of two (**Figure 3B**), an effect relatively moderate. The total lipid content was also affected. Indeed, we observed a 45% increase of the total amount of glycerolipids (estimated through the total FA content; **Figure 3C**), which essentially resulted from a 10–15 times increase of the TAG content (**Figure 3D**). In accordance with this, appearance of lipid droplets was clearly visible using LSC microscopy (**Figure 3E**). The levels of phospholipids showed a tendency to increase, whereas plastidial glycerolipids, i.e., galactolipids and phosphatidylglycerol, were almost not affected (**Figure 3D**). Surprisingly, we did not observe any decline of the amount of PC despite the nearly twofold decrease of the Pi-choline concentration. On the contrary, like most phospholipids, PC showed a tendency to increase. These results indicate that the arrest of cell division following MTX treatment was not accompanied by a significant membrane breakdown, and that the resulting increase in lipid content was largely due to TAG synthesis. It is likely that in such a situation where the expansion of membranes was strongly reduced, the remaining level of Pi-choline was sufficient to supply the required PC turnover. Alternatively, it is also possible that the down-regulation of PC turnover due to the MTX treatment was partly responsible for the growth limitation, thus balancing PC turnover with growth and membrane expansion.

In separate control experiments, we confirmed that these MTX effects specifically resulted from the decline of THF production (Loizeau et al., 2007, 2008) since addition of a folate derivative, 5-formyl-THF (5-FTHF), together with MTX, almost completely reversed the effects observed with MTX (Supplementary Figure 2). It is interesting to note that despite the presence of 5-FTHF, the methyl index (AdoMet/AdoHcy ratio) remained low compared to the control (5.6 versus 40, Supplementary Figure 2B). This result indicates that an AdoMet/AdoHcy ratio of 5.6 is high enough to sustain the methylation reactions required for normal growth. From this point of view, methyl index values of 5–10 have already been reported as control values for *Arabidopsis* cells (Loizeau et al., 2008) and pea leaves (Van Wilder et al., 2009).

**FIGURE 4:**
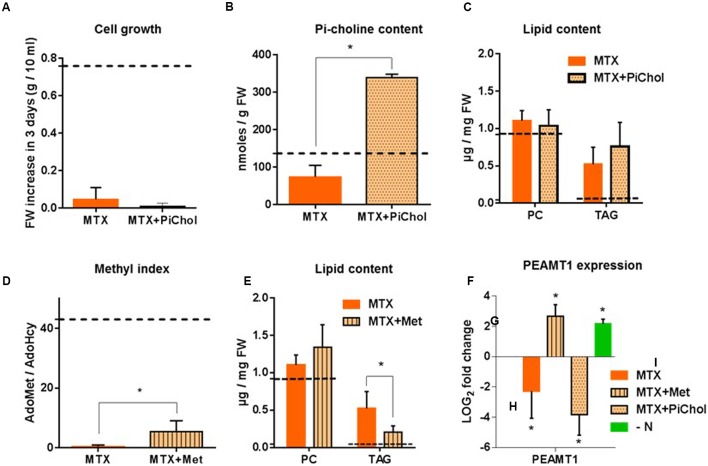
**Attempts to reverse the MTX effects by addition of Pi-choline or Met.** Cells were collected and analysed after 72 h of treatment. **(A)** Comparison of the fresh weight at 72 h when cells were grown in the presence of MTX or MTX + Pi-choline. The initial cell concentrations at *t* = 0 h were 0.3 g per 10 mL of culture in all conditions. **(B)** Intracellular concentration of Pi-choline. **(C)** PC and TAG content in MTX and MTX + Pi-choline treated cells. **(D)** AdoMet/AdoHcy ratio. **(E)** PC and TAG content in MTX and MTX + Met treated cells. **(F)** Effects of MTX, MTX+Met, MTX+Picholine, and nitrogen deficiency on the relative expression of *PEAMT1*; results are expressed as LOG2 fold change versus the control conditions. The dotted lines **(A–E)** represent the values recorded in control cells. For all data, the values are means ± SD of four (MTX) and three (all the other conditions) independent biological repeats, except for the measurements of TAG in MTX and MTX+Met where there were five biological repeats. Significant differences (*P* < 0.05) are shown by an asterisk and were calculated by an unpaired multiple *t*-test using GraphPad Prism software.

To counterbalance the decrease of Pi-Choline resulting from MTX treatment, we supplied the cells with Pi-choline. In separate experiments, we verified that the addition of Pi-choline alone did not affect the glycerolipid composition (Supplementary Figure 3A). Then, comparing MTX and MTX + Pi-choline conditions, we observed that growth remained completely blocked (**Figure 4A**). Despite a higher intracellular Pi-choline concentration (about five times more than in the presence of MTX alone; **Figure 4B**), the level of PC remained roughly constant and TAG still accumulated (**Figure 4C**). These results indicate that the presence of Pi-choline cannot reverse the MTX-induced accumulation of TAG in *Arabidopsis* cells, and that varying the Pi-choline concentration by a factor of five did not significantly affect PC homeostasis.

To explore the possible role of the methyl index in the process of PC synthesis and TAG accumulation, we supplied MTX-treated cells with Met in an attempt to avoid the decrease of the AdoMet/AdoHcy ratio. In separate experiments (Supplementary Figure 3B), we verified that the addition of Met alone did not affect the glycerolipid composition. The presence of Met during the MTX treatment partly restored the methyl index (**Figure 4D**), but not at the same level as that in the control (dotted line). Indeed, the AdoMet/AdoHcy ratio shifted from 0.4 (MTX alone) to 5.4 (MTX + Met), a value similar to the one observed in the presence of MTX + 5-FTHF (see Supplementary Figure 2B). In the MTX + Met situation, the level of PC displayed a tendency to increase compared to the level measured in the presence of MTX alone, although this difference was not statistically significant (**Figure 4E**). TAG still accumulated in the presence of MTX + Met, but the final concentration measured after 3 days was significantly reduced compared to MTX alone. Thus, the presence of Met was able to partly reduce the MTX-induced accumulation of TAG, in contrast to what was observed in the presence of Pi-choline, suggesting that the methyl index, but not the amount of Pi-choline, was a factor influencing TAG accumulation in these experiments. *PEAMT1* was reported to control the level of PC through transcriptional and posttranscriptional regulations (Eastmond et al., 2010). *PEAMT1* expression (**Figure 4F**) was downregulated in the presence of MTX or MTX + Pi-choline but upregulated in the presence of MTX + Met, indicating that the expression of this gene was impacted by the methyl index ratio. However, *PEAMT1* expression was also upregulated in nitrogen-deficient cells, a situation where TAG accumulation was the highest, showing that the expression of this gene depends on several factors (Eastmond et al., 2010). From this point of view, a survey through Genevestigator indicates that *PEAMT1* expression is modified in the presence of plant hormones, downregulated in stress conditions (heat shock, drought, presence of pathogens), and, interestingly, upregulated in a nitrogen -deficient situation, which is confirmed by our own results. Altogether, these results indicate that PC homeostasis is tightly controlled in these cells and point out that altering the methyl index in plants can affect TAG accumulation. Although MTX appeared to target not yet identified pathways that are contributing more to the phenotype than the pathways initially thought, MTX-treated cells remain an interesting tool to study the relationships between membrane and storage lipids.

### Lipid Composition in Nitrogen-Deficient Cells

**FIGURE 5:**
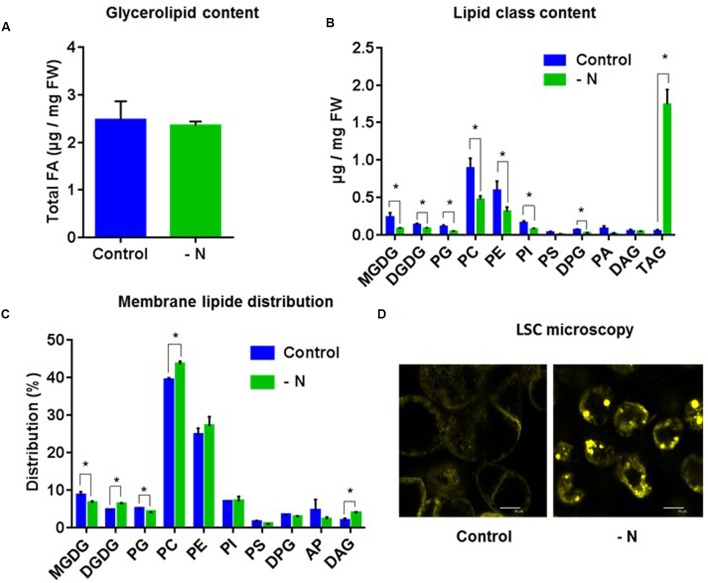
**Lipid analysis of *A. thaliana* cells grown under nitrogen limiting conditions for 72 h. (A)** Total glycerolipid content expressed as μg of FA per mg of fresh weight. **(B)** Quantitative analysis of the various glycerolipids (expressed as μg of FA per mg of fresh weight). **(C)** Membrane lipid distribution: all glycerolipids except TAG. **(D)** Nile-Red staining and LSC microscopy imaging showing an accumulation of lipid droplets in MTX treated cells. The data are means ± SD of three independent biological repeats. Significant differences (*P* < 0.05) are shown by an asterisk and were calculated by an unpaired multiple *t*-test using GraphPad Prism software.

In the second case study, and in contrast with what was observed in the presence of MTX, the total amount of glycerolipids (estimated by the total amount of FA) was not significantly affected (**Figure 5A**), but there was a strong remodeling concerning each glycerolipid class. Indeed, the global content of membrane glycerolipids, phospholipids, and galactolipids, decreased by a factor of two (**Figure 5B**), a decrease seen in all species except DAG. At the opposite, TAG increased 40 times, an accumulation confirmed by the apparition of lipid droplets (**Figure 5D**). Thus, DAG was the only glycerolipid whose concentration remained constant, indicating that this important metabolic hub was tightly regulated. These data strongly suggest that nitrogen-starved cells had a membrane network markedly reduced compared to control cells. However, the distribution of the various membrane lipids (all glycerolipids except TAG) appeared quite robust and did not differ much from the control (**Figure 5C**), except for a slight decrease of MGDG and a slight increase of DGDG and PC that were already observed in leaves from *Arabidopsis* seedlings grown in a nitrogen-deficient medium (Gaude et al., 2007). They also raise, as for the first case study, the question of the origin of TAG. Indeed, were TAG produced *de novo* from three sequential acylation reactions combining glycerol-3-Pi and FA from the acyl-CoA pool (Kennedy pathway), or at the expense of membrane lipids using DAG skeletons issued from phospholipid turnover/breakdown? To further examine this process, we compared in the two case studies the FA composition of the acyl-CoA pool with those of TAG and PC, we measured the expression levels of genes potentially involved in the interconversion of PC, DAG, and TAG, and we undertook PC feeding experiments.

### What Is the Origin of TAG in MTX-Treated and Nitrogen-Deprived Cells?

#### FA Composition

**FIGURE 6:**
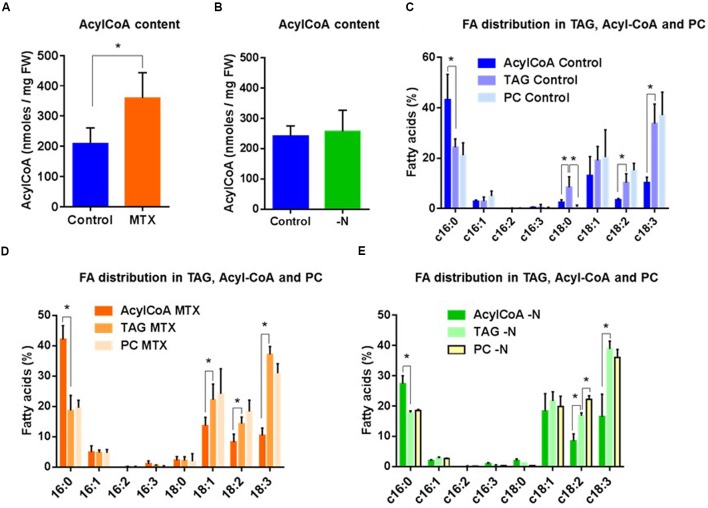
**Fatty acid distribution in acyl-CoA, TAG, and PC pools in MTX-treated and nitrogen-starved cells of *Arabidopsis*. (A)** Impact of the MTX treatment on the total content of the acyl-CoA pool. **(B)** Impact of the nitrogen deficiency on the total content of the acyl-CoA pool. **(C)** FA distribution in acyl-CoA, TAG, and PC in control cells. **(D)** FA distribution in acyl-CoA, TAG, and PC in MTX-treated cells. **(E)** FA distribution in acyl-CoA, TAG, and PC in nitrogen-starved cells. The data are means ± SD of, respectively, five and three independent biological repeats for MTX and –N conditions. Significant differences (*P* < 0.05) are shown by an asterisk and were calculated by an unpaired multiple *t*-test using GraphPad Prism software.

As shown (**Figure 6A**), the acyl-CoA pool was modified by the MTX treatment and increased by nearly a factor of two compared to control cells, reflecting the overall increase of FA previously observed (**Figure 3C**). However, the acyl-CoA pool composition in MTX-treated and control cells were quite different from the one observed in TAG (**Figures 6C,D**). The dominant FA species within the acyl-CoA pool were 16C, whereas 18C represented less than 50% of the total, with 18:1 being the most abundant. In contrast, the dominant FA species in TAG were 18C, representing about 70% of the total, with a marked predominance of 18:3, as it was observed in PC. Noticeably, TAG and PC displayed similar FA compositions, supporting the idea that even in the MTX-induced hyper accumulation of TAG, the PC-DAG pathway could be prevailing over the Kennedy Pathway.

In the nitrogen-deficient situation, the total acyl-CoA content remained roughly similar to the control (**Figure 6B**), here again reflecting the measurements performed on total FA (**Figure 5A**). As for the MTX treatment, the FA composition in TAG did not reflect the acyl-CoA pool, but appeared similar to that of PC (**Figure 6E**). In other words, the FA composition in TAG initially recorded (**Figure 6C**, control cells) never shifted toward the one found in the acyl-CoA pool even after a 40 times increase of the TAG content. This suggests that the large and rapid accumulation of TAG involved edited FA or DAG skeleton originating from membrane lipids (PC), rather than the sequential addition on glycerol-3-Pi of three FA directly withdrawn from the overall acyl-CoA pool (Kennedy pathway).

#### Transcriptomic Analyses of Genes Involved in TAG Synthesis

**FIGURE 7:**
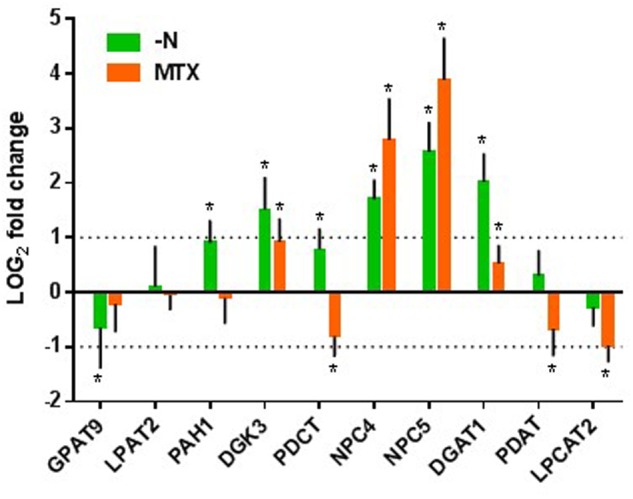
**Effects of MTX and nitrogen deficiency on the relative expression of genes involved in TAG synthesis.** Analyses were made after 72 h of treatment. Results are expressed as LOG2 fold changes versus the control conditions. See **Figure 1** for gene abbreviations. The values between the dotted lines correspond to less than a twofold change. The data are means ± SD of three independent biological repeats. Significant differences (*P* < 0.05) are shown by an asterisk. Statistical analyses were done with GraphPad Prism software using the Holm–Sidak method with α = 5 %.

To further confirm this hypothesis, we looked for the effects of MTX and nitrogen starvation on the expression levels of genes involved in PC-DAG interconversion and TAG synthesis (**Figure 7**; Supplementary Table 1). In nitrogen-deficient cells, the most induced genes were *NPC4*, *NPC5*, and *DGAT1*, which might link membrane lipid turnover with TAG synthesis, as suggested by the above results. Interestingly, the expression of the two first acyltransferases required for the *de novo* synthesis of DAG in the ER, *GAPT9* and *LPAT2*, did not appear to be enhanced in the nitrogen-deficient situation. On the contrary, *GAPT9* displayed a tendency to decrease, although it was by less than a factor of two. Since *GAPT9* was shown to be an essential gene involved in TAG synthesis in oilseeds (Shockey et al., 2016; Singer et al., 2016), this observation further supports the hypothesis of a recycling of lipids rather than only a *de novo* synthesis through the Kennedy pathway. Interestingly, the other genes involved in PC-to-TAG conversion such as *PDCT*, *PDAT*, and *LPCAT* were less impacted than *NPC*, although *PDCT* also showed a tendency to increase.

In the MTX-treated cells, *NPC4* and *NPC5* were the most enhanced. *DGAT1* showed a tendency to increase but varied by less than a factor of two. Since MTX-treated cells accumulated less TAG than nitrogen-deficient ones, it is possible that the initial level of *DGAT1* expression was sufficient to sustain the flux of carbon toward TAG synthesis. *PDCT*, *PDAT*, and *LPCAT2* expressions showed a tendency to be down regulated, highlighting even more the potential role of *NPC* in the TAG production. In contrast with the –N situation, *GAPT9* was not significantly impacted.

#### PC Feeding Experiments in MTX-Treated and N-Deprived Cells

**FIGURE 8:**
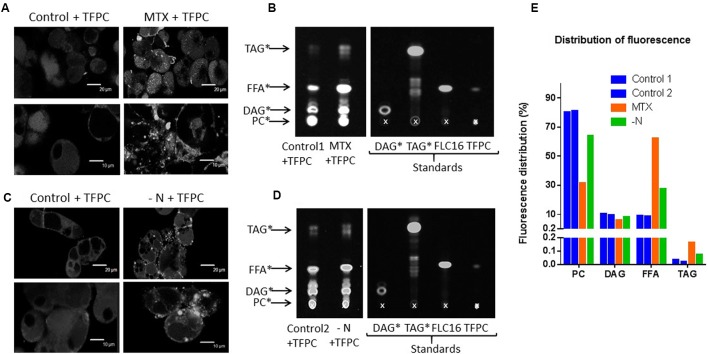
**Fluorescent PC catabolism in control, MTX-treated, and nitrogen-deprived cells. (A,C)** Confocal microscopy of cells incubated for 3 days in the presence of TFPC. Two magnification are shown for both types of cells: 20 μm (upper images) and 10 μm (lower images). The fluorescence of the chromophore was measured at 503 nm following excitation at 488 nm. **(B,D)** Thin layer chromatography (TLC) of neutral lipids showing the effects of MTX and nitrogen starvation on PC to TAG conversion. Control, MTX-treated, and nitrogen-deprived cells, were incubated for 3 days in the presence of 20 nM TopFluor^®^ PC, as described in the “Materials and Methods” section. Then, cells were rinsed to remove the labeled PC before collection and lipid extraction. One hundred micrograms of lipids were deposited on the TLC and run with various standards. Fluorescence was measured at 503 nm following excitation at 488 nm. The standards Bodipy^®^ FL C16 (FLC16), TopFluor^®^ PC (TFPC), Mono-labeled DAG (DAG^∗^), Mono-labeled TAG (TAG^∗^) were prepared as described in the “Materials and methods” section. The cross indicates the initial deposit. **(E)** Distribution of the fluorescence in the different lipid species. Quantification were made from the TLC shown in **(B,D)**, using the ImageJ software.

The strong increase of *NPC4* and *NPC5* expressions in MTX-treated cells suggests a high rate of PC-to-DAG conversion, although there was no apparent decrease of the membrane networks. To verify this point, control, MTX-treated, and nitrogen-deprived cells were incubated for 3 days in the presence of PC having at its *sn*-2 a FA labeled with a fluorescent dye l. As shown in **Figures 8A,C**, control cells mainly displayed a uniform fluorescence pattern presumably representing the membrane network where PC was incorporated. In MTX-treated cells, however, there was a high number of fluorescent dots not seen in control cells, suggesting fluorescent lipid droplet formation. Similar results were observed in cells grown in nitrogen-deficient media. To confirm this assumption, we extracted lipids from these cells and ran a TLC to separate neutral lipids (**Figures 8B,D**). Fluorescent standards were synthesized to identify the lipids present in the extract (see Materials and Methods section). In each condition, the fluorescence present in the extract was quantified and its distribution in the different lipid species was calculated (**Figure 8E**). In control cells, fluorescent PC represented 80% of the fluorescent lipids indicating that it was poorly metabolized. However, it represented only 60 and 30% in the nitrogen-deprived and MTX-treated cells, respectively, suggesting that labeled PC was metabolized more quickly in these two conditions. In cells grown in a media devoid of nitrogen where there is a strong membrane remodeling, it is likely that the externally added PC was in competition for its catabolism with the endogenous PC initially present in membranes (at least in the early hours of starvation). This is a different situation in MTX-treated cells where the production of TAG was not associated with such a membrane breakdown. The fluorescent DAG pool remained almost the same in all situations which might indicate that this pool has reached a steady-state with regard to labeling after 3 days. Although the fluorescent PC was probably not a good substrate for PC metabolizing enzymes, some labeling could also be observed in TAG. Labeled TAG were, respectively, five and four times higher in MTX-treated and nitrogen-deprived cells compared to their respective controls. In both situations, fluorescent free FA were also released from the fluorescent PC, suggesting phospholipase activities. Although previously reported genome-scale transcriptomic studies have shown that most phospholipase A (PLA1 and PLA2) expressions were not significantly affected by a MTX treatment (Loizeau et al., 2008), it remains possible that phospholipases A contributed with NPC to the production of the FA used in TAG formation. Since the fluorescent FA was located at the *sn*-2 position of the glycerol backbone, its accumulation as free FA could also be representative of the editing process. Taken as a whole, these results are in line with the other data reported here and support a higher rate of PC turnover/breakdown in MTX-treated and nitrogen-starved cells, leading to DAG then TAG. Whether TAG were being derived from DAG skeleton released from PC, as suggested by the increased expression of NPC, or from a specific pool of acyl-CoA filled with FA edited from PC, or both, remained an open question.

## Discussion

In the present work, two different situations leading to TAG accumulation were compared and analyzed for their glycerolipid contents and transcriptomic profiles. In the first one, we used the antifolate drug MTX. In the second one, we used nitrogen deprivation, a more physiological situation known to trigger TAG accumulation in plants and microalgae. Although it is not clear about how MTX treatment and nitrogen deprivation result in TAG accumulation, these two different conditions provide useful tools to eventually highlight common answers and to study the relationships between membrane lipids and storage lipids.

### PC Homeostasis

An important observation from our comparative studies lies in the fact that the presence of MTX impaired Pi-choline biosynthesis. This result is supported by a decreased level of *PEAMT* expression, and a decrease by a factor of two of the intracellular Pi-choline concentration. In spite of these, the PC steady-state level remained constant. Similar results were obtained in rat in which MTX treatments induced an increase of TAG in the liver without affecting the PC content (Pomfret et al., 1990). It is clear that in our experimental conditions the membrane expansion is blocked and the need for new PC molecules is limited. However, PC generally displays a dynamic turnover, exchanging acyl chains with DAG and TAG. In particular, several reactions such as those catalyzed by PDCT and NPC involve the interconversion of PC and DAG with the removal or addition of the polar head group. In the presence of MTX, NPC4, and NPC5 [coding for enzymes, respectively, localized in the plasma membrane and in the cytosol (Gaude et al., 2008)] were highly expressed and the rate of PC-to-DAG conversion was enhanced, strongly supporting the idea of a higher turnover between PC and DAG. To maintain PC homeostasis in such circumstances it is likely that a very efficient recycling of the small pool of Pi-choline is required to compensate its reduced rate of production. In all the conditions, we have tested, PC homeostasis was maintained, even in situations which would *a priori* have favored its synthesis, such as in the presence of Pi-choline or Met. In other words, the proportion of PC required in the membrane architecture appeared quite robust, representing in all our experimental conditions about 40 ± 5% of the membrane glycerolipids. Even in nitrogen-deficient cells where the PC concentration decreased two times, it represented about 40% of the membrane lipids since all membrane lipids decreased. How PC synthesis and turnover were coordinated with membrane expansion and cell division remains unclear. It was shown that phosphatidic acid phosphohydrolase (PAH), the enzyme involved in the conversion of PA to DAG, regulates PC synthesis in the ER, and that *pah* mutants displayed a higher expression of *PEAMT1* and a higher rate of PC synthesis (Eastmond et al., 2010). In nitrogen-deficient cells *PAH1* expression slightly increased by less than a factor of two, and in the MTX condition its expression remained unchanged. This suggests that upregulation of *PAH1* was not a key factor in the regulation of TAG accumulation in our experimental conditions.

### TAG Accumulation in Non-seed Organs

Most of the studies leading to our current understanding of TAG synthesis in higher plants were conducted in oilseeds that are specialized organs genetically programmed to accumulate TAG. Mesophyll cell suspension cultures are not known for accumulating oil in standard conditions, and the data described here were metabolic answers to stress situations. The most striking effect observed after MTX addition was a 10- to 15-fold increase in the amount of TAG. This is a specific effect resulting from the blocking of THF synthesis since it was largely reversed by the addition of 5-FTHF, a folate compound often considered as an antidote of MTX. The associated decline of the C1 metabolism has broad effects on cell physiology, including growth arrest due to the impairment of DNA synthesis and perturbation of the methylation reactions, which in turn may affect the regulation of metabolism and gene expressions. Other growth inhibitors such as 5-FU and taxol also triggered TAG accumulation, although not at the same level, indicating some pleiotropic effects. From this point of view, a phenotypic screening with oleaginous microalgae (of the *Nannochloropsis* and *Phaeodactylum* genus) has been recently published (Franz et al., 2013) indicating that a number of very efficient molecules triggering TAG production are primarily growth inhibitors. However, the level of the accumulation is likely linked to the pathway that is targeted. The reversal of MTX-dependent TAG accumulation by the presence of Met suggests, indeed, that part of this accumulation was connected to methyl transfer reactions. The connection between lipids and C1 metabolism is, *a priori*, at the level of PC synthesis which requires several methylation reactions catalyzed by PEAMT or PLMT. However, our attempts to alleviate or bypass the MTX-induced bottleneck of Pi-choline synthesis did not succeed in restoring a normal situation regarding TAG accumulation. Collectively, our data suggest that, in contrast to what is observed in animals, the MTX-dependent accumulation of TAG in higher plants is not directly linked to the inhibition of Pi-choline synthesis.

The regulatory mechanisms that direct carbon fluxes toward either membrane or storage lipids remain to be understood. These two fluxes are probably in competition, the synthesis of membrane lipids (which is connected with cell division) having priority over the synthesis of storage lipids. To date the relationship between cell division and TAG metabolism has been mostly studied in yeast. On nutrient rich media, when yeast cells are actively proliferating, there is a rapid turnover of TAG to release FAs to sustain membrane growth and cell surface expansion (Kurat et al., 2006; Zanghellini et al., 2008; Kohlwein, 2010). In these organisms, TAG synthesis and degradation fluctuate according to the cell division cycle. Inhibiting nucleotide synthesis with MTX should irreversibly block cell division at the G1/S interphase.

**FIGURE 9:**
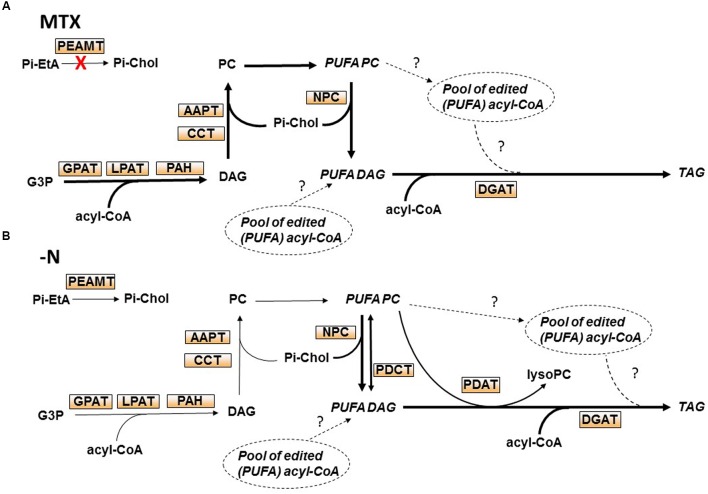
**Putative pathways leading to TAG accumulation in *Arabidopsis* cell cultures during nitrogen deprivation or following MTX treatment. (A)** In the presence of MTX, cell growth and membrane synthesis stopped, and the net increase of FA resulted from TAG synthesis. Because TAG composition was rich in unsaturated FA and similar to the one of PC, we hypothesized that the newly synthesized FA were first incorporated into PC for desaturation. Since *NPC* were strongly enhanced whereas *PDCT, PDAT*, and *LPCAT* were downregulated, we propose that NPC is the main way from PC to TAG. We cannot exclude that a small pool of edited acyl-CoA contributed also to TAG synthesis. Since the level of Pi-choline was reduced in the presence of MTX, but not the level of PC, we hypothesized a strong recycling of Pi-choline. **(B)** During nitrogen deprivation, membrane lipids declined whereas TAG increased, but the total amount of FA remained almost unchanged. Because the FA composition in TAG resembled the one of PC, together with the decrease of *GPAT* expression, we hypothesized that these FA originated more from membrane lipids than *de novo* synthesis. The significant increase of *NPC* and *DGAT* expressions also strongly suggests a PC to DAG to TAG pathway. However, *NPC* was less expressed than in the MTX condition whereas more TAG accumulated, suggesting that other activities such as PDCT and/or PDAT might be involved. Alternatively, a small pool of edited acyl-CoA resulting from the breakdown of membrane lipids could also contribute to the production of TAG. The main routes are shown by wider arrows.

The MTX-dependent TAG accumulation observed here corresponded to a net increase of lipids, with little change in the membrane glycerolipid composition. The situation was the opposite in nitrogen-deficient cells where membrane glycerolipids dropped by a factor of two, whereas the total lipid content did not change much. Thus, TAG represented about 50% of total lipids in these nitrogen-deprived cells. It is likely that in the absence of protein synthesis, cells could not maintain their membrane networks, and that FA normally stored into phospho- and galactolipids were redirected toward storage lipids. It is interesting to note that the FA composition in TAG was always similar to the one found in PC and quite different from the one in the acyl-CoA pool. Indeed, in all conditions the acyl-CoA pool contained more 16:0 and less 18:2 and 18:3 than TAG or PC. Even in situations where TAG were accumulating, the TAG composition remained closer to the one of PC than to the one of acyl-CoA. This strongly suggests that TAG synthesis in these cells preferentially used FA previously edited at the level of phospholipids (PC), an idea also supported by the higher rate of PC turnover observed in the two situations leading to TAG accumulation. From this point of view, we observed in the MTX and nitrogen-deficient conditions a strong increase of *NPC* expressions suggesting a higher activity of non-specific phospholipase C that could provide, together with the PDCT activity (Lu et al., 2009), the DAG skeletons required for this synthesis. *PDCT* expression appeared to be upregulated in the case of nitrogen deficiency whereas it was downregulated in the presence of MTX, which might explain why in the –N condition *NPC* are less expressed despite the fact that TAG accumulated more. The marked increase of *DGAT1* expression in the case of nitrogen deficiency also supports the idea that, at least in this situation, the third FA originated from the acyl-CoA pool. Alternatively, TAG might be produced from a small and specific pool of edited acyl-CoA, not in equilibrium with the main pool. These findings are tentatively summarized in **Figure 9**.

## Conclusion

These results show differences but also similarities between the two TAG accumulating situations. In the MTX condition, the membrane network was maintained and TAG accumulation corresponded to a net increase of lipids. In the nitrogen-deficient situation, the strong TAG accumulation was associated with a decrease of the membrane networks without marked increase of the overall lipid content. In the two situations, the high turnover of PC, the FA composition in TAG, and the high *NPC* expressions highlight a route implying PC-DAG conversion, as it is also the case in *Arabidopsis* seeds (Bates and Browse, 2011; Shockey et al., 2016). A future challenge will be to clarify the role of NPC in the dynamic exchange between PC and DAG during the course of TAG formation in plant cells, and to determine whether this role could be generic or specific to a series of metabolic reprogramming in response to environmental stresses or cell division effectors.

## Author Contributions

CM, MC, RH, FB, and FR performed the experiments. CM, EM, and FR designed, supervised, and analyzed the experiments. All authors contributed to the writing of the article.

## Conflict of Interest Statement

The authors declare that the research was conducted in the absence of any commercial or financial relationships that could be construed as a potential conflict of interest. The reviewer DVDS declared a past collaboration with one of the authors FR to the handling Editor, who ensured that the process nevertheless met the standards of a fair and objective review.
