# Supplementary material for: C1 Metabolism Inhibition and Nitrogen Deprivation Trigger Triacylglycerol Accumulation in Arabidopsis thaliana Cell Cultures and Highlight a Role of NPC in Phosphatidylcholine-to-Triacylglycerol Pathway
Source: Front Plant Sci. 2017 Jan 4;7:2014. doi: 10.3389/fpls.2016.02014 (PMC5209388; doi:10.3389/fpls.2016.02014)
Supplement: Supplementary file 1 [file Data_Sheet_1.DOCX]

Supplementary Material

**C1 metabolism inhibition and nitrogen deprivation trigger triacylglycerol accumulation in *Arabidopsis thaliana* cell cultures and highlight a role of NPC in phosphatidylcholine-to-triacylglycerol pathway**.

**Coline Meï, Mathilde Cussac, Richard Haslam, Frédéric Beaudoin, Yung-Sing Wong, Eric Maréchal and Fabrice Rébeillé^*^**

*** Correspondence: Corresponding Author:** fabrice.rebeille@cea.fr

**1 Supplementary Figures and Tables**

**1.1 Supplementary Tables**

**Supplementary Table 1**: Expression of genes involved in TAG and PC syntheses in cells cultivated in presence of MTX, MTX + Met and MTX + Pi-choline. Analyses were performed after 72h of treatment. RT-qPCR data in each condition are expressed as fold change versus the respective controls (no MTX). The values showing more than a twofold change versus controls are in bold. nd=not determined. The data are means ± SD of five biological repeats for the MTX condition and three for the other conditions. Statistical analyses were performed with GraphPad Prism software using unpaired t test and the Holm-Sidak method with α = 5%.

|  | | MTX | | MTX + Met | | MTX + Pi-chol | | - N | |
| --- | --- | --- | --- | --- | --- | --- | --- | --- | --- |
| Protein | Atg number | Avg. | *StD.* | Avg. | *StD.* | Avg. | *StD.* | Avg. | *StD.* |
| NPC4 | AT3G03530 | **7.77*** | *3.84* | **56.54*** | *51.41* | **5.77*** | *2.49* | **3.33** | *0.77* |
| NPC5 | AT3G03540 | **16.69*** | *9.20* | **48.31*** | *26.41* | **5.72*** | *2.69* | **5.83** | *2.60* |
| PDAT | At5g13640 | 0.66* | *0.22* | **0.48*** | *0.24* | nd |  | 1.28 | *0.37* |
| DGAT1 | At2g19450 | 1.47* | *0.38* | 0.82 | *0.32* | 0.72 | *0.65* | **4.01** | *1.63* |
| DGAT2 | AT3G51520 | **0.42*** | *0.31* | nd |  | nd |  | **2.19** | *0.71* |
| LPCAT1 | AT1G12640 | 1.45 | *0.86* | nd |  | nd |  | 1.18 | *0.33* |
| LPCAT2 | At1g63050 | 0.52* | *0.10* | 0.52 | *0.28* | nd |  | 0.84 | *0.17* |
| PDCT | At3g15820 | 0.59* | *0.14* | 1.27 | *1.02* | nd |  | 1.76 | *0.37* |
| PEAMT1 | At3g18000 | **0.35*** | *0.28* | **7.15*** | *3.50* | **0.09*** | *0.08* | **4.59** | *0.99* |
| PEAMT2 | At1g48600 | **0.48** | *0.36* | 1.89 | *0.97* | nd |  | nd |  |
| PEAMT3 | At1g73600 | **0.45*** | *0.25* | **2.30*** | *0.55* | **0.44*** | *0.38* | **0.29** | *0.11* |
| AAPT1 | At1g13560 | 0.74 | *0.46* | 0.59* | *0.29* | 0.50 | *0.44* | nd |  |
| CCT1 | At2g32260 | 0.70 | *0.35* | 0.83 | *0.36* | **0.18*** | *0.16* | nd |  |
| PLMT | At1g80860 | 1.19 | *0.72* | 0.73 | *0.38* | **0.30*** | *0.26* | nd |  |
| GPAT9 | AT5G60620 | 0.91 | *0.32* | nd |  | nd |  | 0.71 | *0.33* |
| LPAT2 | AT3G57650 | 0.98 | *0.18* | nd |  | nd |  | 1.18 | *0.55* |
| PAH1 | AT3G09560 | 0.97 | *0.27* | 0.64* | *0.25* | nd |  | 1.92 | *0.49* |
| DGK1 | AT5G07920 | 1.1 | *0.6* | 0.61 | *0.22* | nd |  | nd |  |
| DGK3 | AT2G18730 | 1.95* | *0.5* | 0.72* | *0.22* | nd |  | **3.1** | *1.3* |
| DGK7 | AT4G30340 | 1.49 | *1.3* | 1.82* | *0.38* | nd |  | nd |  |

**Supplementary Table 2**: Primer sequences used for RT-qPCR analysis of the genes involved in PC and TAG syntheses in Arabidopsis cells.

|  |  | Primer sequence 5' - 3' | |
| --- | --- | --- | --- |
| Gene name | Atg number | Forward | Reverse |
| *PEAMT1* | AT3G18000 | TGAGCACTGGTGGACTTGAGACAA | GCTGAGTCCAATAGCACGTTCCA |
| *PEAMT2* | AT2G44170 | ATG GGT CAA GCC AGG GGG AT | GCC CCA ATG CAT TTG CAG CCA |
| *PEAMT3* | At1G73600 | CTTTGACGTGGATGTTGTGG | TGGTGCAATCAGCTACTTCG |
| *CCT1* | AT2G32260 | ATGCTGCGCAACGAGTGGGT | TCTCCAGCCTCTCTGCCGACTT |
| *AAPT1* | AT1G13560 | TTTACCGCCACCGGACCATCTG | GCGTTATCATGTTTGGTGGCATCC |
| *PLMT* | AT1G80860 | AATGCTCGTGCCAAATCCATCTCA | TCCAGTTGGTTCTTTGCAGCAGATG |
| *NPC4* | AT3G03530 | GTGATAGGTGGTTTGCGTCGGT | AAGAGAGTGGAAGGAGGGAATTGGT |
| *NPC5* | AT3G03540 | GCCTGGCACAGTTCTTCATGGG | CCGGATTTGACAATGTTTCAGGGCA |
| *PDCT* | AT3G15820 | TCGCGTATTAGCATCTTCACCGGA | GAAAGTCCTGAGGGAGAGGAAGCTG |
| *DGAT1* | AT2G19450 | AGTCCACTTAGCTCCGACGCA | TGAAAAGCGGCCAATCTCGCA |
| *PDAT* | AT5G13640 | GCGTGGTGCCGCTCATTTCT | TGCAACTGTCGGGAGACTGGT |
| *LPCAT2* | AT1G63050 | ACTCATCCGTCGGTTTCATGGTTTT | AGTAGCTGAGAAGAAGCACAGCGA |
| *GPAT9* | AT5G60620 | CCACAAACCATAAGGCCCGGTG | TGCGAAACTCTGTTGCTTGCGT |
| *LPAT2* | At3G57650 | AGTTGACTGGTGGGCTGGAGTT | GCAACCTGACCGCTGAGCC |
| House keeping genes | |  |  |
| *ACTINE 7* | AT5G09810 | ATGGAACTGGAATGGTGAAGGC | GGACGACCAACAATACTTGGGAAC |
| *ARP1* | AT1G43170 | AGGTTGGTACTGAGGCACACAC | AGTCACATCCTTCTCAGTCCTGTC |
| *UBIQUITINE 5* | AT3G62250 | ATAATCTTCAGCAGCCGTTGCC | ACCTCAAGGGTTATGGTCTTCCC |

**1.2 Supplementary Figures**

**Supplementary Figure 1**: Representative growth curves showing the effect of the various inhibitors used in Fig2. Arabidopsis cells were cultured in 100 ml flasks and the growth of the population was followed for 3 days through the increase of the fresh weight. After 3 days (end of the exponential phase of growth for control cells), cells were collected for analyses.


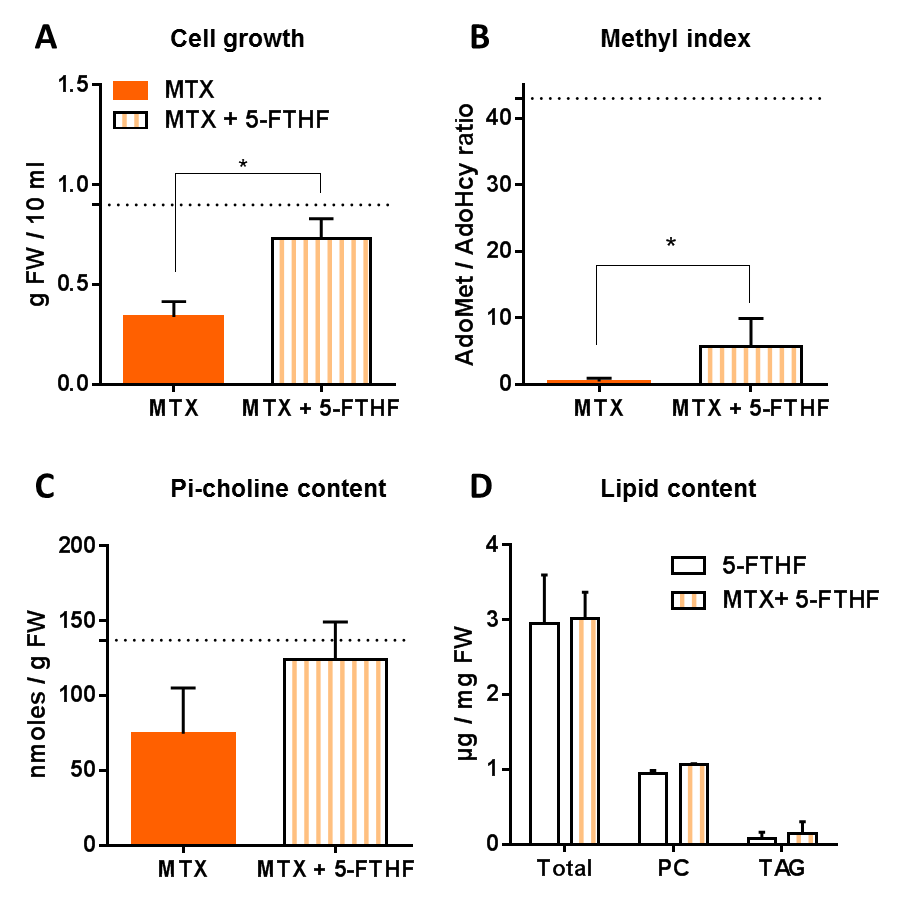
**Supplementary Figure 2**: Attempts to reverse the MTX effects by 5-Formyltetrahydrofolate (5-FTHF). Cells were collected and analysed after 72h of treatment. A, Fresh weight at 72h. The initial cell concentrations at t=0h were 0.3 g per 10mL of culture in all conditions. The upper dotted line represents the fresh weight of the control condition after 72h. B, AdoMet / AdoHcy ratio. C, Pi-choline concentration. D, Total glycerolipid, PC and TAG contents expressed as µg of FA per mg of fresh weight. The data are means ± SD of three different biological repeats. Statistical analyses were done with GraphPad Prism software.

**Supplementary Figure 3**: Effect of Pi-choline (A) and Met (B) on the glycerolipid distribution in Arabidopsis cells. Cells were collected and analysed after 72h of treatment. Each result is the average of at three biological repeats ± SD.
